# Prevalence of mental disorders in migrants compared with original residents and local residents in Ningxia, China

**DOI:** 10.1186/s12888-016-1088-y

**Published:** 2016-10-28

**Authors:** Zhizhong Wang, Liqun Wang, Jinyun Jing, Chunping Hu

**Affiliations:** 1 Department of Epidemiology and Statistic, School of Public Health, Ningxia Medical University, Yinchuan, 750004 China; 2 Department of Psychiatry, Minkang Psychiatric Hospital of Civil Affairs, Ningxia Yinchuan, 750010 China; 3 1160#, Shengli Street, Yinchuan, 750004 China

**Keywords:** Ecological migrants, Mental disorders, Epidemiology, Mainland China

## Abstract

**Background:**

Ecological migrants has a special background compared with other types of migrant. However, the mental health status of ecological migrants who were expected to benefit from a massive “ecological migration project” initiated by the Chinese government is unknown. This study aims to explore the influence of environmental change on individuals’ mental health and to improve current understanding of the mechanisms that mental disorders occurred.

**Methods:**

The data were extracted from a cross-sectional study. Anxiety disorders, mood disorders and substance use disorders were assessed using the Chinese version WHO-CIDI. The prevalence of mental disorders was stratified by migration status into ecological migrant, local resident and original resident groups. Unconditional logistic regression models were used to calculate the risk of prevalence among these three groups.

**Results:**

After controlling for gender, ethnicity, age, marriage, and education, the migrants had lower risk of mental disorders than original residents [OR = 0.70 (95 % CI: 0.57–0.86)], *p* < 0.001), but had a higher risk of mental disorders than local residents [OR = 1.29 (95 % CI: 1.06–1.55)], *p* = 0.007).

**Conclusion:**

The ecological migration project may be beneficial to people’s mental health by improving their living environment and social economy.

## Background

The migration experience can be difficult at best. Migrants are exposed to a variety of difficult situations in the destination country [1, 2]. Additionally, migrants may have had traumatic experiences in their places of origin, as some are refugees from warzones, international laborers or individuals in exile [3]. Most studies have shown that the migration experience can increase the risk of psychiatric disturbances over both the short and the long term [4]. For example, greater psychological distress and psychiatric morbidity have consistently been found among migrants from the former Soviet Union compared to Israel-born natives [5, 6]. These negative influences can persist for generations [7, 8]. Some studies have indicated that the influences of migration on mental health depends on the motivations of migration. For example, people who immigrate to unite family members have been shown to have fewer mental disorders than refugees who are forced to leave their countries because of war, severe weather or natural disasters [9].

In general, migration refers to the process of leaving one’s country to live in another, and the word migrant refers to those persons. Hundreds of thousands of studies have focused on migrants who left their homes in one country and traveled to another to take up residence. Only a few studies, however, have focused on migrants who relocated within the same country, such as rural-urban migrant workers [10], ecological migrants (ecomigrants), and project migrants [11] (such as the Three Gorges Dam Project, the world’s largest hydroelectric dam built in 1994 which flooded or partially flooded thirteen cities, and forced millions of residents to leave their hometowns in areas submerged by the reservoir). Ecological migration is caused by environmental deterioration (e.g., desertification and drought) that forces people to leave their homes; once relocated, these persons must adjust their lifestyles to those of the local population [12]. There are approximately 25 million ecomigrants in the world, taking into account both international and internal migration. In Bangladesh, an estimated 12 million to 17 million people have fled their homes in recent decades because of environmental disasters – those living in low-elevation areas are likely to experience more intense flooding in the future, forcing them to leave their homelands. In several countries in Africa’s Sahel region, which borders the Sahara, approximately 10 million people have been driven to move because of droughts and famines [13]. Compared with traditional migrants, ecological migrants in Ningxia have a unique history, culture, and religious background [14]. The province of Ningxia is located in western China, and over one-third of the population is Hui ethnicities. The Hui ethnic minority group is largely descended from those who came to China from Saudi Arabia seeking work. Consequently, this ethnic group is composed almost entirely of Muslims [15, 16]. In the 1990s, the government began to help residents in southern mountainous areas (in which seven counties were identified as project counties by the Ningxia Development and Reform Commission) resettle in the northern plain region of Ningxia. As of 2011, a total of 630,000 residents who amount to approximately 30 % of the total population of the project counties were involved in the migration projects in Ningxia [17].

The field of migrant epidemiology examines the association of migration with the rate of disease occurrence [18]. Studies of this type can help clarify whether a disease of unknown causes is principally determined by genetic inheritance or by environmental exposure. For example, migration from a high-risk population to a low-risk population should not affect the occurrence of a genetically determined disease among migrants. In contrast, migration from a high-risk population to a low-risk population is expected to be associated with a reduction in the occurrence of an environmentally determined disease.

The current study aimed to examine the risk of mental disorders in ecological migrants compared with local residents and original residents to explore the association of migration with mental disorders; and to improve the current understanding of the mechanisms that mental disorders occurred. We hypothesized that ecological migrants, who were expected to benefit from the massive “ecological migration project” initiated by the Chinese government, had a lower risk of mental disorders than that of original residents.

## Methods

### Data sources

The data analyzed in this study were obtained from Epidemiological Survey of Mental Disorders in Ningxia (ESMD-NX). The sampling process has been described elsewhere [19]. First, 62 primary sampling units (PSUs) were selected from 2,602 primary units (include 2209 villages and 393 neighborhood communities) using a probability proportional to size method. Second, depending on the total number of households in the selected PSUs, 60 to 210 households were systematically identified from each PSU, yielding 6,890 selected households. Third, interviewers visited each household and used the Kish selection table to identify one eligible participant from each household based on the inclusion criteria (aged 18 years or older and a resident at the current address for at least 6 months) and the exclusion criteria (unconsciousness caused by brain injury, brain tumor and/or craniotomy or dementia; being in the acute phase of a cerebrovascular accident; experiencing a severe illness that prevents communication; having any obvious cognitive disabilities; or currently suffering from deafness, aphasia or other language barriers). A total of 414 households were excluded because a participant could not be located during the study period, resulting in a sample of 6,476 residents participated in the interview. Those with missing data were excluded, yielding a total of 5,811 participants (89.7 %) who completed the full interview used for further analysis.

This analysis includes 4,366 participants. Of these, 1,726 were ecological migrants, 1,458 were local residents (those who lived in the northern plain region located along the Yellow River, which is more developed than the southern mountainous areas of Ningxia, with per capita gross domestic production (GDP) over 5,000 US dollars in 2012 and offers convenient transportation and a better living environment), and 1,182 were original residents (indigenous people who have long lived in the southern ecologically fragile mountainous areas, where receive scant rainfall, have poorly nourished land and with per capita GDP was less than 1,500 US dollars in 2012). We identified ecological migrants through the following questions: “How many times have you moved to a totally new village?” If the response was “one or more times,” they were asked two more questions: “Did you move to this village because of the government’s Ecological Migration Project?” and “How many years have you lived in this village?” We defined the ecological migrant group as those who had moved, were involved in the Ecological Migration Project, and had lived at their current address for at least 2 years. Participants sampled from the project counties and lived in rural areas were defined as the original residents group. Participants who sampled from counties located in the plain region of Ningxia (where the ecological migrants were resettled) and lived in rural areas were defined as the local group.

### Measurements

In-person CAPI [20] was conducted by trained lay medical college students from July 2011 to January 2013. The 12-month prevalence of anxiety disorders (including agoraphobia, generalized anxiety disorder, obsessive-compulsive disorder, panic disorder, social phobia, specific phobia, and neurasthenia), mood disorders (including unipolar depressive disorder and bipolar disorder), and substance use disorders (including alcohol use disorders and tobacco dependence) were determined. The WHO Composite International Diagnostic Interview (WHO-CIDI) [21] was used to diagnose mental disorders according to the ICD-10 diagnostic criteria. “Any mental disorder” was defined as those who had experienced at least one of the mental disorders mentioned above.

Sociodemographic information was collected using the demographic section of the WHO-CIDI, including age, gender, education, marital status, ethnicity (Han vs. Hui), and history of migration from other areas of the province (yes vs. no). Physical health variables included self-reported type II diabetes (yes vs. no) and hypertension (yes vs. no).

### Data analysis

The analyses were performed using Statistical Analysis System (SAS) software version 8.2 (SAS Institute Inc., Durham NC, USA.). The prevalence of anxiety disorders, mood disorders and substance use disorders was stratified by migration status into ecomigrant, local resident and original resident groups. Post-stratification adjustment weight was applied when estimating the prevalence among three groups, and gender (male and female), age (<30, 30–40, 40–50, 50–60, and over) and ethnicity (Hui and Han) were used in constructing the post-stratification adjustment according to the Sixth Census of Ningxia 2010. The Rao-Scott chi-square test was used to assess differences in weighted prevalence among three groups [22]. Differences in sociodemographic characteristics and physical health variables among migrants, local residents, and original residents were examined using one-way analysis of variance (ANOVA) for continuous variables and chi-square test for categorical variables. Four separate non-conditional logistic regression models were created to estimate the risk of mental disorders across the three groups for different mental disorders under controlling for demographic variables. Two dummy variables were created to specify the groups being compared (group_a : 1 = local residents, 0 = migrants + original residents; group_b : 1 = original residents, 0 = migrants + local residents). Odds ratios, along with their 95 % confidence intervals, were calculated for all models. Given the exploratory nature of these analyses, the statistical significance level was set at 0.05.

## Results

### Demographic characteristics of the participants

As shown in Table 1, the participants’ demographic characteristics varied across the three groups. The average age of the migrants was significantly younger than that of the local and original residents (*P* < 0.001). The proportion of women in the migrant group was higher than that in other groups (*P* < 0.001). Additionally, the migrant group had a higher proportion of Hui individuals.

**Table 1:** Demographic characteristics of the participants

Variables	Migrant *N* = 1726	Local *N* = 1458	Original *N* = 1182	*F/x* ^*2*^	*P*
Age (year, mean, SD)	39 (15)	47 (14)	44 (15)	111.27	<0.001
Education (year, mean, SD)	4.5 (4.3)	5.2 (4.1)	5.2 (5.1)	13.05	<0.001
Gender (male,%)	38.9	42.4	47.5	21.30	<0.001
Ethnicity (Hui,%)	57.0	26.3	48.6	313.8	<0.001
Marriage (%)					
married	87.6	90.6	89.0	61.49	<0.001
divorced or widowed	2.5	5.5	2.9		
unmarried	9.8	3.8	8.1		
Current smoking (%)	14.3	23.2	24.0	55.17	<0.001
Diabetes mellitus (%)	1.3	2.6	1.8	6.89	0.032
Hypertension (%)	8.6	15.0	13.1	32.57	<0.001

### The comparison of the twelve-month prevalence of mental disorders among the three groups

In Table 2, the original residents with higher prevalence of mental disorders (15.9 %) than migrants (13.6 %) and local residents (10.3), *P* < 0.001. Similar results for the anxiety disorder. Meanwhile, the migrant had higher prevalence of mood disorder than original residents and local residents.

**Table 2:** The prevalence of mental disorders among migrants, local residents and original residents, % (weighted prevalence)

Variables	Migrant *N* = 1726	Local *N* = 1458	Original *N* = 1182	*x* ^*2*^	*P*
Any mental disorder	15.3 (13.6)	10.5 (10.3)	16.3 (15.9)	22.07	<0.001
Anxiety disorder	11.8 (10.0)	7.4 (7.0)	12.2 (11.7)	9.46	0.008
Mood disorder	2.7 (2.7)	1.2 (0.9)	2.4 (2.4)	21.38	<0.001
Substance use disorder	0.6 (0.9)	1.2 (1.3)	1.4 (1.6)	3.22	0.199

### Multivariable analysis

As shown in Table 3, controlling for demographical variables and physical health variables, migrants had a higher risk of any mental disorders than local residents, which also was true for anxiety and mood disorders. And migrants with a lower risk of any mental disorders, as well as of anxiety disorders, than original residents. Additionally, no significant differences in the risk of substance use disorders were found among the three groups.

**Table 3:** Odds ratios estimated by logistic regression

Variables	B (SE)	*P* value	aOR (95 % CI)
Any mental disorder (*N* = 4366)			
age	0.02 (0.04)	0.677	1.02 (0.94,1.10)
gender	0.22 (0.09)	0.017	1.25 (1.04,1.51)
ethnicity	0.37 (0.09)	<0.001	1.44 (1.20,1.74)
education	−0.02 (0.01)	0.045	0.98 (0.95,1.00)
divorced/widowed	−0.12 (0.25)	0.638	0.89 (0.55,1.45)
unmarried	0.25 (0.19)	0.175	1.29 (0.89,1.86)
Diabetes mellitus	0.11 (0.30)	0.713	1.12 (0.61,2.03)
hypertension	0.18 (0.14)	0.202	1.20 (0.91,1.58)
local residents	−0.35 (0.10)	<0.001	0.70 (0.57,0.86)
original residents	0.25 (0.09)	0.007	1.29 (1.06,1.55)
Anxiety (*N* = 4366)			
age	0.05 (0.04)	0.223	1.05 (0.97,1.15)
gender	0.59 (0.11)	<0.001	1.81 (1.45,2.26)
ethnicity	0.45 (0.11)	<0.001	1.57 (1.27,1.94)
education	−0.03 (0.01)	0.012	0.97 (0.94,0.99)
divorced/widowed	−0.16 (0.27)	0.552	0.85 (0.49,1.46)
unmarried	0.44 (0.21)	0.041	1.55 (1.02,2.37)
Diabetes mellitus	0.19 (0.33)	0.552	1.22 (0.64,2.31)
hypertension	0.21 (0.16)	0.186	1.23 (0.90,1.67)
local residents	−0.41 (0.12)	<0.001	0.66 (0.52,0.83)
original residents	0.26 (0.10)	0.014	1.30 (1.05,1.61)
Mood disorders (*N* = 4366)			
age	0.13 (0.09)	0.148	1.14 (0.95,1.36)
gender	0.25 (0.23)	0.277	1.28 (0.82,2.00)
ethnicity	0.17 (0.22)	0.451	1.18 (0.76,1.83)
education	−0.01 (0.03)	0.891	0.99 (0.94,1.05)
divorced/widowed	0.58 (0.46)	0.211	1.78 (0.72,4.40)
unmarried	0.35 (0.43)	0.405	1.43 (0.62,3.29)
Diabetes mellitus^a^	−13.98 (765.2)	0.985	-
hypertension	−0.21 (0.36)	0.561	0.81 (0.40,1.64)
local residents	−0.84 (0.28)	0.002	0.43 (0.24,0.74)
original residents	0.18 (0.22)	0.672	1.20 (0.77,1.88)
Substance use disorders (*N* = 4364)			
age	−019 (0.13)	0.142	083 (0.64,1.07)
gender^a^	−14.64 (184.30)	0.937	-
ethnicity	−0.70 (0.34)	0.040	0.49 (0.25,0.97)
education	0.01 (0.04)	0.762	1.01 (0.94,1.09)
divorced/widowed^a^	−12.50 (533.20)	0.981	-
unmarried^a^	−14.22 (430.20)	0.974	-
Diabetes mellitus	1.45 (0.66)	0.028	4.25 (1.17,15.45)
Hypertension	−0.18 (0.51)	0.721	0.83 (0.30,2.28)
local residents	0.07 (0.31)	0.824	1.07 (0.57,2.00)
original residents	0.36 (0.31)	0.245	1.44 (0.77,2.66)

^a^No proper ORs were calculated because very few positive cases report substance use disorders in one of the group

*SE* standard error, *OR* odds ratio, *95 % CI* 95 % confidence interval, *aOR* adjusted odds ratia

## Discussion

Over the past few decades, migration has become policy in some areas of developing countries. Moreover, migration is an important health issue, as changes in the physical and social environment may associated with disease patterns [23–25]. With the support (include family based housing, community based road repairing and developing public transport) of the Ningxia government, people living in adverse ecological areas and those living in poor counties (districts) have relocated with family to better living conditions. Original residents lived in the less developed mountainous areas, while local residents lived in the well-developed plain area of Ningxia.

This study found that the risk of mental disorders significantly differed among migrants, local residents, and original residents. One possible reason is that the migration is often motivated by economic, education, age, gender, among other characteristics. Additionally, gender and ethnicity may be risk factors for any mental disorders and anxiety. Local residents have higher socioeconomic status than original residents, which may benefit migrants. Ecological migrants were significantly younger than both local residents and original residents, with a higher proportion of females. Those demographic differences may contribute to the lower prevalence of mental disorders among migrants compared with original residents. The present study found when controlling for demographics, migrants were consistently less likely to have mental disorders than original residents, but they were at a higher risk of mental disorders than local residents. The finding is consistent with previous studies [26].

Ecological migration is often perceived as a means to a better end, i.e., to lifting oneself and one’s family out of poverty and improving one’s standard of living [27]. Income level may affect physical conditions, and a high income may have a positive association with mental health. It should be noted that economic, educational and social support are accessible to ecological migrants in China. Migrants may have experienced better mental health in this study because of the improvements in their living conditions and the financial support provided by the Chinese government (include offer allowance, reduce tax, and living skills training), but these benefits do not eliminate the potentially negative influences of previous experiences in their original homes on mental health. Previous studies have shown that workers who migrate from rural to urban settings in China are not particularly vulnerable to poor mental health. This may be because of the sense of well-being that is associated with upward economic mobility and improved opportunities or because of the relatively high social capital found in migrant communities [28]. This finding is consistent with research conducted outside of China as well. For example, Patel reviewed 11 community-based studies and found that most of them reported an association between indicators of poverty and the risk of mental disorders [29]. Similarly, Nandi et al. found a dramatic decrease in anxiety disorders among those with improved living conditions in a 20-year cohort study [30].

The Ecological Migration Project has been a key environmental policy in which thousands of people have participated, especially in the northern areas of China. This study is the first to explore the association of migration with the mental health in ecomigrants. These findings may further the current understanding of how environmental and genetic factors influence the development of mental disorders.

This study has several limitations. First, given its cross-sectional design, this study cannot identify causal relationships between migration and the mental disorders. Second, this study does not examine the relationship between mental disorders and participants’ decisions or motivations to migrate from their homes and, thus, from their social resources. There were few positive cases for every single specific disorders, the present study failure to evaluate the influence of migration on the specific mental disorders. Prospective data will be needed to help identify the causal factors that influence the development of mental disorders in this population.

## Conclusions

This study showed that after adjust the influence of gender, ethnic, age and other factors, the migrants more likely had mental disorders than local residents, and had a lower prevalence than original residents. The findings indicate that ecological migration project in Ningxia area did not increase the burden of mental disorders of the immigrant population, due to the cross-sectional design, further prospective studies needed to verify the conclusions.

## Abbreviations

CAPI: Computer-assisted personal interview
GDP: Gross domestic production
PSU: Primary sampling units
WHO-CIDI: World health organization composite international diagnostic interview
